# *MEPE* loss-of-function variant associates with decreased bone mineral density and increased fracture risk

**DOI:** 10.1038/s41467-020-17315-0

**Published:** 2020-10-23

**Authors:** Ida Surakka, Lars G. Fritsche, Wei Zhou, Joshua Backman, Jack A. Kosmicki, Haocheng Lu, Ben Brumpton, Jonas B. Nielsen, Maiken E. Gabrielsen, Anne Heidi Skogholt, Brooke Wolford, Sarah E. Graham, Y. Eugene Chen, Seunggeun Lee, Hyun Min Kang, Arnulf Langhammer, Siri Forsmo, Bjørn O. Åsvold, Unnur Styrkarsdottir, Hilma Holm, Daniel Gudbjartsson, Kari Stefansson, Aris Baras, Regeneron Genetics Center, Xiaodong Bai, Suganthi Balasubramanian, Leland Barnard, Andrew Blumenfeld, Michael Cantor, Giovanni Coppola, Aris Economides, Gisu Eom, Lukas Habegger, Young Hahn, Alicia Hawes, Marcus B. Jones, Shareef Khalid, Luca A. Lotta, Evan K. Maxwell, Lyndon J. Mitnaul, John D. Overton, Jeffrey G. Reid, Manuel Allen Revez Ferreira, William Salerno, Deepika Sharma, Alan Shuldiner, Jeffrey C. Staples, Ashish Yadav, Goncalo R. Abecasis, Kristian Hveem, Cristen J. Willer

**Affiliations:** 1 Division of Cardiovascular Medicine, Department of Internal Medicine, University of Michigan, 1500 E. Medical Center Dr., Ann Arbor, MI 48109 USA; 2 Department of Biostatistics and Center for Statistical Genetics, University of Michigan School of Public Health, 1415 Washington Heights, 1700 SPH I, Ann Arbor, MI 48109 USA; 3 Program in Medical and Population Genetics, Broad Institute of Harvard and MIT, 415 Main Street, Cambridge, MA 02142 USA; 4 Department of Computational Medicine and Bioinformatics, University of Michigan, Palmer Commons, 100 Washtenaw Avenue, Ann Arbor, MI 48109 USA; 5 Regeneron Genetics Center, 777 Old Saw Mill River Road, Tarrytown, NY 10591 USA; 6 K.G. Jebsen Center for Genetic Epidemiology, Department of Public Health and Nursing, NTNU, Norwegian University of Science and Technology, NO-7491 Trondheim, Norway; 7 MRC Integrative Epidemiology Unit, University of Bristol, Oakfield House, Oakfield Grove, Bristol, BS8 2BN UK; 8 Clinic of Thoracic and Occupational Medicine, St. Olavs Hospital, Trondheim University Hospital, Prinsesse Kristinas gate 3, 7030 Trondheim, Norway; 9 HUNT Research Centre, Department of Public Health and Nursing, Norwegian University of Science and Technology, Postboks 8905, N-7491 Levanger, Norway; 10 Department of Endocrinology, St. Olavs Hospital, Trondheim University Hospital, Prinsesse Kristinas gate 3, 7030 Trondheim, Norway; 11 deCODE genetics/Amgen, Inc., Sturlugata 8, 101 Reykjavik, Iceland; 12 School of Engineering and Natural Sciences, University of Iceland, Sturlugata 7, 101 Reykjavik, Iceland; 13 Faculty of Medicine, University of Iceland, Vatnsmýrarvegur 16, 101 Reykjavik, Iceland; 14 Department of Human Genetics, University of Michigan, 4909 Buhl Building, 1241 E. Catherine St, Ann Arbor, MI 48109 USA

**Keywords:** Computational biology and bioinformatics, Predictive medicine, Genetics, Genetic association study, Genome-wide association studies

## Abstract

A major challenge in genetic association studies is that most associated variants fall in the non-coding part of the human genome. We searched for variants associated with bone mineral density (BMD) after enriching the discovery cohort for loss-of-function (LoF) mutations by sequencing a subset of the Nord-Trøndelag Health Study, followed by imputation in the remaining sample (*N* = 19,705), and identified ten known BMD loci. However, one previously unreported variant, LoF mutation in *MEPE*, p.(Lys70IlefsTer26, minor allele frequency [MAF] = 0.8%), was associated with decreased ultradistal forearm BMD (*P*-value = 2.1 × 10^−18^), and increased osteoporosis (*P*-value = 4.2 × 10^−5^) and fracture risk (*P*-value = 1.6 × 10^−5^). The *MEPE* LoF association with BMD and fractures was further evaluated in 279,435 UK (MAF = 0.05%, heel bone estimated BMD *P*-value = 1.2 × 10^−16^, any fracture *P*-value = 0.05) and 375,984 Icelandic samples (MAF = 0.03%, arm BMD *P*-value = 0.12, forearm fracture *P*-value = 0.005). Screening for the *MEPE* LoF mutations before adulthood could potentially prevent osteoporosis and fractures due to the lifelong effect on BMD observed in the study. A key implication for precision medicine is that high-impact functional variants missing from the publicly available cosmopolitan panels could be clinically more relevant than polygenic risk scores.

## Abstract (web summary)

Bone mineral density (BMD) is associated with fracture risk and many genetic loci with small effect sizes have been discovered by genome-wide association studies (GWAS). Here, the authors discover a large-effect rare loss-of-function genetic variant for BMD in the *MEPE* gene in the Norwegian HUNT study which replicates in the UK Biobank.

## Introduction

The mineral content of bone reaches peak during young adulthood; as humans age, the mineral content of bone decreases and porosity increases, weakening the bones and leaving them vulnerable to fracture. Measurements of the density of bones, typically determined by x-ray absorption, can predict which individuals are at risk of hip, vertebral and other fractures but are often performed clinically only after a fracture occurs^1^. Bisphosphonate and other oral, subcutaneous and intravenous medications can be prescribed to increase bone mineral density (BMD) and reduce fracture risk in osteoporotic individuals^2^. BMD is typically measured in individuals with high risk of osteoporosis (for example, due to family history of osteoporosis, use of corticosteroids or use of antiestrogen in breast cancer treatment) and is recommended to be tested after a fracture.

BMD is a complex trait with a strong genetic component; heritability estimates range between 50 and 85%^3–6^ and genome-wide association studies demonstrate this is mostly through polygenic effects. Additionally, there are multiple forms of monogenic skeletal diseases caused by single mutations^7^, but these variants are typically very rare (<1/1000). Genome-wide studies of estimated BMD, as measured by ultrasound of heel have identified almost 900 associated genomic regions^8–10^, with a substantial number also associated with fracture. Genomic discovery can aid in identifying targets for novel therapeutics, and potentially for identification of individuals at-risk for fracture that may benefit from preventive therapies.

Human diseases have typically been studied by testing associations between human genetic variation and phenotypes, where the discovered variants and genes are often investigated experimentally in model organisms or cell-based systems that can be genome-edited or perturbed in the laboratory. On the one hand, studying genetic mutations in humans themselves provides the natural genetic background and environmental conditions that lead to disease, but we are limited to observing the genetic changes that have arisen spontaneously in the human genome over time, and the frequency spectrum of variants that can be tested is limited by technology, cost and presence of those variants in the population under study. While on the other hand, the study of animal models can often provide conflicting or uninterpretable results when applied to humans, sometimes resulting in expensive clinical trials that fail.

To advance the translation of genetic discovery to improved therapeutics and prevention via prediction of at-risk individuals, we sought to identify rare and low-frequency loss-of-function (LoF) variants associated with BMD and fractures through a genome-wide association study (GWAS). We employed methodology wherein we performed low-pass sequencing of a subset of the sample (*N* = 2202), then imputed variants, including insertion/deletion polymorphisms, into the remainder of the HUNT discovery sample (*N* = 19,705) followed by replication of previously unreported variants in two independent replication samples: UK Biobank (*N* = 279,435) and deCODE (*N* = 170,000). Using this approach, we identify a LoF mutation in *MEPE*, which may be useful for precision medicine and therapeutic development.

## Results

### Genome-wide screen for BMD-associated LoF variants

The Nord-Trøndelag Health Study (HUNT)^11^ performed screening of BMD during enrollment into the HUNT study at different time points: HUNT2 in 1995–1997 and HUNT3 in 2006–2008. The standard technology in use at that time was single-energy x-ray absorptiometry (SXA), and the decision was made to focus on ultradistal forearm BMD measurements. Although this is not the current standard used in clinic or hospital-based cohort collections, the HUNT study has the advantage of a population-based screening of individuals with a wide variety of ages, with the inclusion of healthy individuals relative to a clinic-based phenotype, and decades of longitudinal clinical follow-up including fractures. Furthermore, it has been demonstrated that the T-score derived from wrist BMD is correlated with hip T-score (*r* = 0.61) and lumbar T-score (*r* = 0.53)^12^, suggesting that ultradistal forearm BMD is helpful for estimating risk of fractures as well as diagnosing osteoporosis^13^.

**Fig. 1:**
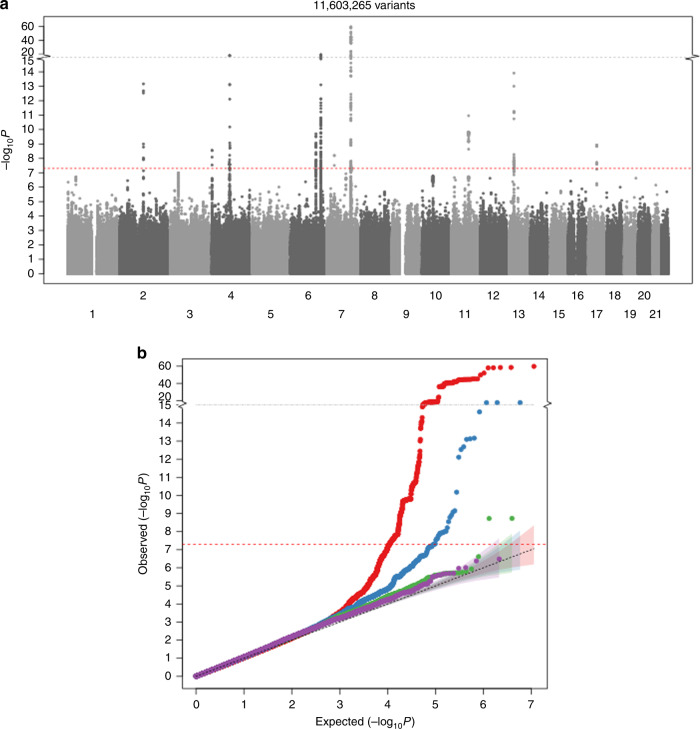
Ultradistal forearm BMD Genome-wide association analysis results. Manhattan (upper panel) and QQ-plot (lower panel) in HUNT dataset (*N* = 19,705) for ultradistal forearm bone mineral density (BMD) genome-wide association analysis. In the Manhattan plot (upper panel) the genome-wide significance threshold (*P*-value < 5 × 10^−8^) is shown using a red dotted line. In the QQ-plot (lower panel), the tested variants have been divided into four groups based on MAF (red dots = MAF [0.05; 0.5], blue dots = MAF [0.005; 0.05], green dots = MAF [0.001; 0.005], purple dots = MAF [0.000253; 0.001]). MAF: minor allele frequency. −log_10_P: −1 × tenth logarithm of the association test *P*-value.

To enrich the discovery cohort for rare loss-of-function (LoF) variants typically missed by array-based genotyping, we first performed low-pass whole genome sequencing (*N* = 2202, on average 5X coverage) followed by imputation into the remaining HUNT samples, and tested for association with 11.2 M single nucleotide variants and 430,000 indels with high imputation quality (imputation *R*^*2*^ > 0.9) and minor allele count >10 in 19,705 samples. We replicated 10 previously identified BMD loci with genome-wide significant associations with ultradistal forearm BMD (association test *P*-value < 5 × 10^−8^, Fig. 1, Table 1). One of the BMD-associated loci, *MEPE* on chromosome 4, spanned over a 5 Mb window and contained the lead intergenic variant reported for association with femoral neck and lumbar spine BMD by the GEFOS consortium in 2009 (rs1471403^14^) as well as the lead variant from the UK Biobank estimated heel bone mineral density (eBMD) GWAS^10^ (rs11934731; *r*^*2*^ = 0.71 with GEFOS lead variant). In the HUNT discovery cohort (*N* = 19,705), the minor allele at the lead single nucleotide variant at this locus, rs181831514, had a much higher impact (effect = −0.53 SD [standard deviation units], minor allele frequency [MAF] = 0.8%), was much less common than the previously identified lead variants and was in nearly perfect linkage disequilibrium (*r*^*2*^ = 0.999) with a rare LoF indel in the *MEPE* gene (rs753138805, *MEPE* p.Lys70IlefsTer26, *P*-value = 2.1 × 10^−18^). The insertion/deletion polymorphism was only observed following imputation from HUNT low-pass sequences which included indel calling. The Haplotype Reference Consortium imputation panel (which contains 1254 HUNT low-pass sequences which we submitted) was able to impute the intronic proxy variant (rs181831514; imputation *R*^*2*^ = 0.99) but the indel was not present. The 1000 Genomes reference panel, which does include indel calls does not have p.Lys70IlefsTer26 present; however, the proxy variant imputation quality (imputation *R*^2^) from 1000 Genomes reference dataset was 0.9979.

**Table 1:** Genome-wide significant loci in the discovery HUNT sample.

rsID	Chromosome	Position (hg19)	Effect allele/non-effect allele	Annotation (candidate gene)	Effect allele frequency	Imputation quality (*R*^*2*^)	Effect size^a^ (SE)	Association test *P*-value
rs115242848	2	119507607	T/C	Intergenic (*EN1*)	0.011	0.966	0.387 (0.052)	6.9 × 10^−14^
rs4505759	4	1003022	T/C	Upstream (*FGFRL1*)	0.304	0.996	0.069 (0.012)	2.8 × 10^−9^
rs181831514	4	88822746	T/C	Intergenic (*MEPE*)	0.008	0.988	−0.533 (0.061)	2.1 × 10^−18^
rs7741021	6	127468274	C/A	Intronic (*RSPO3*)	0.474	0.998	0.068 (0.011)	2.0 × 10^−10^
rs4869742	6	151907748	T/C	Intronic (*CCDC170*)	0.273	0.992	−0.108 (0.012)	2.4 × 10^−19^
rs6973667	7	38152863	G/A	Intergenic (*STARD3NL*)	0.337	0.981	0.066 (0.011)	6.2 × 10^−9^
rs2707518	7	120954908	T/G	Intergenic (*WNT16*)	0.367	0.989	0.182 (0.011)	1.7 × 10^−60^
rs489247	11	86881641	G/A	Intronic (*TMEM135*)	0.258	0.997	−0.083 (0.012)	1.1 × 10^−11^
rs2147161	13	42982302	C/A	Intergenic (*TNSFS11*)	0.701	0.957	−0.091 (0.012)	1.2 × 10^−14^
rs76410205	17	41807508	T/C	Intergenic (*SOST*/*DUSP3*)	0.096	0.971	0.111 (0.018)	1.2 × 10^−9^

This table shows the 10 genome-wide significant (*P*-value < 5 × 10^−8^) loci associated to ultradistal forearm bone mineral density (BMD) in the discovery dataset (*N* = 19,705). As all these are previously known loci, the candidate gene has been taken from the previous publications. The effect of a variant is presented with the SAIGE linear mixed model effect size (Effect size) and standard error (SE) and the significance using the uncorrected two tailed Z-test *P*-value.

*rsID* reference SNP cluster ID, *SE* standard error of the effect estimate.

^a^Measured in SD units.

### Statistical evidence for *MEPE* p.Lys70IlefsTer26

*MEPE* p.Lys70IlefsTer26 is located only 65 kb away from the previously identified association lead variant from a previous GWAS for eBMD in white British individuals^8^ (LD *r*^*2*^ = 0.06). The nominally significant association signal at this lead variant (rs11934731, effect size for the minor allele = 0.029 SD, *P*-value = 0.01, MAF = 33%) was slightly attenuated when we performed conditional analysis with *MEPE* p.Lys70IlefsTer26 as a covariate in the model (effect size_conditional_ = 0.021 SD, *P*-value_conditional_ = 0.06; Supplementary Fig. 1, Supplementary Data 1). Additionally, the association for the *MEPE* LoF variant remains highly significant when conditioning for the lead variant of the eBMD analysis (effect size_conditional_ = −0.529 SD, *P*-value_conditional_ = 5.3 × 10^−18^).

The LoF deletion demonstrated a very strong association with ultradistal forearm BMD (effect size = −0.53 SD, *N* = 19,705, *P*-value = 2.1 × 10^−18^, Table 2) and has an eight fold stronger impact on BMD than the common variant previously reported as associated with femoral neck and lumbar spine BMD by the GEFOS Consortium^14^ at this locus (effect size = −0.07 SD, MAF = 0.34). We replicated this finding in exome sequence data from 279,435 UK Biobank individuals with estimated heel BMD (eBMD), which demonstrated a genome-wide significant association with a similar effect size (effect size = −0.48 SD, *N* = 279,435, *P*-value = 1.2 × 10^−16^, Table 2) as observed in Norwegian HUNT individuals. The proportion of UK individuals carrying at least one copy of *MEPE* p.Lys70IlefsTer26 was nearly 17 fold lower (0.095%) than the frequency observed in Norwegian HUNT individuals (1.6%). Additionally, this mutation shows comparable effect size (effect size for dual energy x-ray absorptiometry [DXA] arm BMD = −0.588, *N* = 15,092) in the Icelandic population where, however, the variant is even more rare (MAF = 0.03%), and thus lacks the power to replicate the association (*P*-value = 0.12).

**Table 2:** Association of the *MEPE* LoF variant to BMD phenotypes in the three study datasets.

Dataset	Frequency of the deletion allele	Phenotype	Effect for the deletion allele (SD units)	SE	*N*	Association test *P*-value
HUNT	0.8%	Ultradistal forearm BMD	−0.53	0.061	19,705	2.1 × 10^−18^
UK Biobank	0.05%	Estimated heel BMD	−0.48	0.059	279,435	1.2 × 10^−16^
deCODE	0.03%	Whole body BMD	−0.62	0.372	14,194	0.10
deCODE	0.03%	Hip (femoral neck) BMD	−0.33	0.201	34,486	0.10
deCODE	0.03%	Arm BMD	−0.59	0.374	15,092	0.12
deCODE	0.03%	Lumbar Spine BMD	−0.07	0.214	33,746	0.76

This table shows the association results for all three datasets (HUNT, UK Biobank and deCODE) and all tested bone mineral density phenotypes for the *MEPE* loss-of-function frameshift deletion, p.Lys70IlefsTer26 (rs753138805, chr4: 88766219 GAAA/-). The effect of a variant is presented with the effect size (Effect size) and standard error (SE) and the significance using the uncorrected two tailed Z-test *P*-value.

*BMD* bone mineral density, *SD* standard deviation, *SE* standard error of the effect estimate, *N* number of samples.

### *MEPE* LoF variant clinical characterization

To determine the impact of *MEPE* p.Lys70IlefsTer26 on clinical end points, we performed association analyses for bone-related phenotypes in HUNT (Supplementary Data 2) in the full HUNT dataset with genetic information available (*N* = 69,716). *MEPE* p.Lys70IlefsTer26 carriers show higher risk for multiple types of fractures as well as osteoporosis with odds ratios (OR) ranging between 1.35 and 2.06 (Table 3). We see similar ORs in the Icelandic replication dataset, but the only significant association is for the forearm fractures of old individuals (Supplementary Table 1). In the UK population we see nominally significant association to any fracture (OR = 1.76 [1.00; 3.11], Supplementary Table 1).

**Table 3:** Significant PheWAS results for the *MEPE* LoF mutation in HUNT dataset.

Description	OR [95% CI]	Association test *P*-value	#cases/#controls
Fracture of ankle and foot	1.83 [1.42; 2.35]	3.3 × 10^−6^	5478/45480
Fracture of upper limb	1.51 [1.26; 1.82]	1.2 × 10^−5^	11128/45480
Any fracture	1.35 [1.18; 1.54]	1.6 × 10^−5^	24155/45480
Fracture of radius and ulna	1.61 [1.29; 2.00]	1.8 × 10^−5^	7998/45480
Fracture of foot	2.06 [1.48; 2.86]	2.0 × 10^−5^	3223/45480
Osteoporosis	1.58 [1.27; 1.97]	4.2 × 10^−5^	6994/61558
Osteoporosis, osteopenia and pathological fracture	1.50 [1.22; 1.84]	1.1 × 10^−4^	8077/61558
Senile osteoporosis	1.69 [1.28; 2.22]	1.8 × 10^−4^	4482/61558
Fracture of humerus	2.01 [1.39; 2.90]	2.0 × 10^−4^	2457/45480
Fracture of unspecified bones	1.46 [1.19; 1.79]	3.4 × 10^−4^	8627/45480
Fracture of hand or wrist	1.52 [1.19; 1.94]	7.7 × 10^−4^	5860/45480

This table presents all significant (*P*-value < 1.2 × 10^−3^, Bonferroni correction for 42 phenotypes) end-point associations for the *MEPE* LoF frameshift deletion in HUNT dataset (*N* = 69,716). The effect of a variant is presented with the odds ratio (OR) and 95% confidence intervals (CI) and the significance with uncorrected two tailed Z-test (for log(OR)) *P*-value. Full phenome-wide association scan (PheWAS) results and ICD codes underlying the phenotypes can be found from Supplementary Data 2.

*OR* odds ratio, *CI* confidence interval.

Typical diagnostic criteria for osteoporosis is a BMD more than 2.5 SD below the reference population average (T-score < −2.5). As we did not have the official T-score available for the dataset, which is always in relation to a reference dataset BMD distribution, we used the ultradistal forearm BMD lower than −2.5 SD as a proxy. Within the HUNT participants with genotypes and BMD measured (*N* = 19,705), the proportion of individuals who experienced any fracture during an average of 31 years of follow-up was 41.0% (*N* = 8082)—this includes all types of fractures and all causes including trauma. Within the relatively small subset of individuals with ultradistal forearm BMD < −2.5 SD at the time of BMD measurement, 53.3% had experienced a fracture (65 of 122; Supplementary Table 2). Similarly, almost half (49.0%) of the individuals that carried the p.Lys70IlefsTer26 mutation had experienced a fracture (149 of 304, OR = 1.39 [1.11; 1.74]) during the follow-up period, which was not significantly different from those with low BMD (BMD < −2.5 SD, OR = 1.65 [1.15; 2.35]).

To compare the potential clinical impact of this single variant, we calculated a polygenic score for eBMD in the HUNT dataset based on Kim et al.^8^ results using 1032 independently associated variants and weights from the UK Biobank cohort (Supplementary Data 3). Of the HUNT individuals in the lowest 1% of the BMD polygenic score distribution (i.e., 1% of the population with highest genetic burden for low BMD, fractures and osteoporosis), 38.2% experienced fracture during the follow-up, which is not significantly different from the rate in the remaining individuals (OR = 0.89 [0.66; 1.19], Fisher test *P*-value = 0.46, Supplementary Table 2). We performed the same comparison for the 5% and 10% tails, and similarly saw no difference in the fracture rates (OR = 0.98 [0.86; 1.12], Fisher test *P*-value = 0.79 and 1.04 [0.94; 1.14], Fisher test *P*-value = 0.44, respectively).

**Fig. 2:**
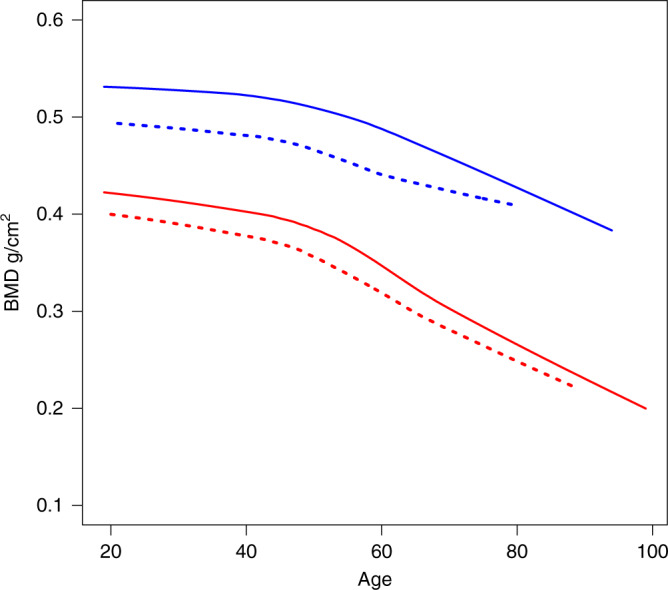
Age trend in BMD for *MEPE* LoF mutation carriers. In this figure we have compared the forearm bone mineral density (BMD) in the *MEPE* loss-of-function (LoF) mutation, p.Lys70IlefsTer26, carriers (dotted lines) compared to non-carriers (solid lines) in the HUNT dataset (*N* = 19,705). The trend over different ages is illustrated using LOWESS curve for males (blue lines) and females (red lines) separately.

When examining the decrease in ultradistal forearm BMD by age in both LoF carriers and non-carriers, we can see that the loss of BMD in both subgroups is the same (Fig. 2) suggesting that *MEPE* p.Lys70IlefsTer26 mutation affects the BMD peak value, rather than the lifetime bone mass loss similarly to the *LGR4* stop-gain variant previously identified by Styrkarsdottir et al.^15^. In our dataset, the ultradistal forearm BMD of an average 20 year-old woman carrying the LoF mutation in *MEPE* has a similar ultradistal forearm BMD as a 54 year-old non-carrier woman. A 20 year-old man carrying the LoF mutation in *MEPE* had an ultradistal forearm BMD similar to the average BMD for a 64 year-old male carrier (Supplementary Fig. 2).

## Discussion

By sequencing followed by imputation into a large population-based study in Norway, we identified a LoF mutation in the *MEPE* gene, p.Lys70IlefsTer26, that demonstrates a genome-wide significant and high-impact association with ultradistal forearm BMD and with increased risk of osteoporosis and fractures. Common variants at this locus have been associated with BMD-related traits in previous studies^10,14^, but we were able to pinpoint this association to a rare LoF mutation in the gene, definitively establishing a causal role and direction of effect of *MEPE* on BMD.

Matrix extracellular phosphoglycoprotein (*MEPE*) was first cloned as a candidate for the oncogenic phosphaturic factor with similarities to a group of bone–tooth mineral matrix phospho-glycoproteins^16^. *MEPE* is expressed mainly in bone marrow and brain bone-associated tumors^16^. Mice with an ablated *MEPE* gene displayed increased osteoblast number, osteoblast activity and a higher bone mass^17^, whereas *MEPE* overexpression in bone inhibited bone growth and mineralization in mice^18^. Although how *MEPE* regulates osteoblast activity remains unclear, *MEPE* expression shows different patterns during osteoblast differentiation in human and mice. In mice, Mepe increases with differentiation^19^, while in human *MEPE* expression peaks in the proliferation phase and is suppressed during further differentiation^20^.

*MEPE* p.Lys70IlefsTer26 is the second rare LoF mutation found to be associated with BMD in population-based genome-wide association studies. A study by Styrkarsdottir et al.^15^ found a rare stop-gain mutation in *LGR4* gene with higher effect than the *MEPE* frameshift mutation (effect size for whole body BMD = −0.75 SD, *P*-value = 1.6 × 10^−6^, *N*  =  7359) that was associated with a binary phenotype for low hip, spine or whole body BMD (defined as < −1.0 SD, *P*-value = 1.3 × 10^−10^). The same research group has also identified two rare missense mutations in *COL1A2* associated with low BMD^21^ in an Icelandic dataset with *N* = 209,379 (p.Gly496Ala; MAF = 0.1%, effect size for low spine BMD < −1SD = 1.53, *P*-value = 1.8 × 10^−7^ and p.Gly703Ser; MAF = 0.05%, effect size for low hip BMD < −1SD = 2.23, *P*-value = 1.9 × 10^−8^). A study by Zheng et al.^22^ identified non-coding mutations with MAF~1% in *EN1* and *WNT16* genes using whole-genome sequence data from the UK10K cohort (*N* = 2882), followed by imputation into over 20,000 samples. Their results demonstrated a 4-fold increase in the effect size for the low-frequency variants compared to the common variants found in the previous GWAS.

Because the mutation status can be determined at birth, *MEPE* LoF carriers may benefit from treatment to preserve or increase peak BMD prior to the age of peak BMD. As the carriers show a similar rate of BMD loss during the adulthood as non-carriers, it may be sufficient for these individuals to be treated during the late childhood and early adulthood to increase the peak BMD. The promise of possible prevention of fractures by screening the population for *MEPE* mutations relative to measuring BMD is that *MEPE* p.Lys70IlefsTer26 carriers can be identified and provided treatment prior to decrease of BMD, which has the potential to prevent fractures in this subgroup and perhaps maintain higher lifetime BMD. Also, *MEPE* LoF carrier status identifies twice as many at-risk individuals as the BMD < −2.5 SD criterion in HUNT study participants (1.54% carry LoF vs 0.62% have BMD < −2.5 SD). We suggest that current clinical practice could be augmented with additional screening for the carriers of LoF variants of the *MEPE* gene. The finding that carriers of the mutation had lower ultradistal forearm BMD even during young adulthood, when bone mass would be expected to peak, suggests that these individuals may benefit from early initiation of osteoporosis prevention.

There are some limitations to our study. We acknowledge the difference between the discovery dataset and replication phenotypes, ultradistal forearm BMD measured with SXA (HUNT), compared to DXA from lumbar spine, hip, arm and whole body in deCODE and heel bone BMD measured with ultrasound in the UK biobank. However, as can be seen from Supplementary Fig. 3, the correlation between effect estimates for these two phenotypes is fairly high (0.7–0.8) when comparing SNPs with adequate power in the smaller dataset. Additionally, as we have hospital registry data from the Nord-Trøndelag county only, it is possible that some of the HUNT study participants have experienced a fracture that is not accounted for in our dataset.

Mendelian randomization studies have demonstrated that BMD is a causal risk factor for fracture^23^. Therefore, we suggest that screening for individuals at high genetic risk could aid in starting appropriate pharmaceutical therapies and avoid fracture risk in these individuals. We performed low-pass whole genome sequencing of 2202 individuals followed by imputation into ~20,000 individuals from the HUNT study of Norway. We did not deeply sequence the *MEPE* gene in all Norwegians in this study, suggesting that additional LoF variants in this gene may be observed. By identifying other *MEPE* LoF mutations carriers, on top of the 1.6% with the p.Lys70IlefsTer26, we could increase the number of individuals who could be protected from fracture caused by low BMD. In addition to the European population, the *MEPE* LoF variant is present in African and Latino populations but with an extremely low allele frequency. However, given the presence of 16 different LoF mutations in the UK population^24^, different LoF mutations may be present, but as-yet-undetected in other populations.

This study demonstrates that continued investigation of genetic variation in humans, particularly rare variants identified through sequencing, can identify genetic variants that clearly and immediately define functional genes and may be useful for precision medicine and therapeutic development.

## Methods

### HUNT genotype dataset

The discovery dataset, The Nord-Trøndelag Health Study (HUNT)^11^, is a population-based cohort of ~120,000 (descriptives in Supplementary Table 3) from the county of Nord-Trøndelag, Norway. Since 1984, phenotype data has been collected for these individuals approximately every 11 years. Participation in the HUNT Study is based on informed consent and the study has been approved by the Data Inspectorate and the Regional Ethics Committee for Medical Research in Norway (REK: 2014/144). In total, approximately 70,000 HUNT individuals have been genotyped using the Illumina Human CoreExome v1.1 array from both HUNT2 (collected between 1995–1997) and HUNT3 (collected between 2006 and 2008). After quality control of the genotype data, 69,716 European ancestry samples were imputed using a combined Haplotype Reference Consortium reference panel and a population specific whole genome sequence-based imputation panel^25^. 11.2 M single nucleotide variants and 430,000 indels with high imputation quality (imputation *R*^*2*^ > 0.9) and minor allele count >10 were included in the analysis. Variants were annotated as a LoF mutation (3510 variants) if predicted as LoF (stop gain, stop loss, splice variant or frameshift) for either UCSC, Ensembl or RefSeq transcripts by ANNOVAR in Whole Genome Sequence Annotator^26^ v0.7.

### HUNT phenotypes

BMD (in g/cm^2^) was measured at the ultradistal part of the non-dominant forearm by single-energy x-ray absorptiometry (DTX100; Osteometer MediTech, Inc, Hawthorne, CA), and the measurements were standardized using equipment-specific correction factors^27^ estimated by three repeated hydroxyapatite bone imitation measurements of the European Forearm Phantom (QRM GmbH, Moehrendorf, Germany). BMD was measured in a subset of adult HUNT participants, including: 5% random samples of all participants, random samples of female participants in selected municipalities and age-groups within 35–85 years of age, and participants reporting ever having asthma, asthma-related symptoms or use of asthma medication (detailed selection criteria are available at ntnu.edu/hunt/databank). The present analyses were restricted to participants of European ancestry. For individuals who had their BMD measured in either HUNT2 collection, HUNT2 follow-up (2001) or HUNT3 collection, the HUNT2 measurement was prioritized, follow by HUNT3, then HUNT2 follow-up. The final discovery analysis dataset consisted of 19,705 samples with both imputed genome information and BMD.

Using the unique 11-digit national identification number that is allocated to all Norwegian citizens, we linked the HUNT study data to prospectively recorded information on fractures at the hospitals serving Nord-Trøndelag county: the local Levanger and Namsos Hospitals (Nord-Trøndelag Hospital Trust) and St. Olavs Hospital, Trondheim University Hospital. ICD-9 and ICD-10 codes from the electronic patient administrative systems were available from all hospitals from September, 1987 through October, 2017. For forearm and hip fractures at Levanger and Namsos Hospitals from October, 1995 through December, 2012, all diagnoses were validated by examination of medical records (relevant ICD codes accompanied by a procedure code for reduction, surgical intervention, or intervention with a rigid device), confirmation by X-ray or by review by a medical doctor^28^. A full list of PheCodes (derived from ICD-9 and ICD-10 codes) included in the phenome-wide association analysis can be found in Supplementary Data 2.

### Statistical methods for discovery in HUNT

The association analysis in the discovery dataset was performed using SAIGE^29^, which implements linear or logistic mixed effects model (for quantitative and binary phenotypes respectively) accounting for sample relatedness and subtle population structure. The association analyses for inverse normal transformed ultradistal forearm BMD and clinical end points were adjusted with age (birth year for the clinical end points), sex, the first 4 genetic principal components and genotyping batch. Formal conditional analysis for the *MEPE* locus was performed using the same software, model and covariates as the discovery association analysis by adding the LoF variant as an additional covariate in the linear mixed model. Due to power restrictions, the analyses for clinical end points were restricted to PheCode-derived diagnoses with at least 500 cases. The sample size for the ultradistal forearm BMD association analysis was *N* = 19,705 and for the clinical end-point analyses *N* = 69,716. Clinical end points reaching *P*-value < 1.2 × 10^−3^ (Bonferroni correction for 42 end points) were regarded as statistically significant. The LOWESS (Locally Weighted Scatterplot Smoothing) curve for age trend was fitted using smoother span (the proportion of points in the figure affecting the local value) of 2/3. The Fisher tests for comparing different predictors for fractures and all the Figures have been done using R (https://cran.r-project.org) v3.5.3.

### Replication datasets

Replication of the association at the *MEPE* LoF variant, p.Lys70IlefsTer26, was tested within the UK Biobank whole-exome sequence dataset in 279,435 participants. All participants in the UK Biobank provided informed consent and the study has obtained Research Tissue Bank (RTB) approval from its ethics committee (The Research Ethics Committee approval number; 11/NW/0382). Detailed cohort descriptions, sequencing, imputation and analysis methods for the UK biobank replication dataset can be found from Van Hout et al.^24^. Briefly, 302,342 participants (of which 279,435 with eBMD) were exome sequenced (coverage exceeds 20X at 95.5% of sites on average) resulting in ~12 million variants in targeted regions. Heel bone quality was evaluated with two methods; quantitative ultrasound speed of sound and broadband ultrasound attenuation using a Sahara Clinical Bone Sonometer (Hologic Corporation, Bedford, Massachusetts, USA)^10^. Raw values for eBMD were first stratified by sex, rank-inverse normal transformed, and then re-combined. Association analysis was performed using a linear mixed model implemented in BOLT-LMM v2.3.2 (https://data.broadinstitute.org/alkesgroup/BOLT-LMM/) with covariates for age, age-squared, and first ten genetic principal components. The replication analysis for fractures (ICD codes S22–S92, excluding skull [S02] and neck [S12]) was ran using SAIGE with age, age^2^, age–sex interaction, sex and first ten genetic principal components as covariates.

The Icelandic replication dataset (deCODE)^30,31^ is based on 170,000 genotyped samples which have been imputed using a whole-genome sequenced population specific imputation panel. All participating individuals, or their guardians, gave their informed consent before blood samples were drawn and the study has been approved by the National Bioethics Committee and the Icelandic Data Protection Authority. Using these samples, the genotypes of 375,984 samples have been imputed using familial imputation. The imputation quality for the *MEPE* p.Lys70IlefsTer26 (imputation info score) was 0.99. The BMD in the dataset has been measured using DXA from lumbar spine, hip, arm and whole body. Additionally, the dataset has health-care registry data available, which have been used in the end-point association replication.

## Supplementary information

Supplementary Information

Peer Review File

Description of Additional Supplementary Files

Supplementary Data 1

Supplementary Data 2

Supplementary Data 3

## Data Availability

The GWAS summary statistics are available at http://csg.sph.umich.edu/willer/public/bmd2020/. All other data that support the findings of this study are available from the corresponding author upon reasonable request. The Haplotype Reference Consortium imputation panel is accessible through the Michigan Imputation Server (https://imputationserver.sph.umich.edu/index.html#!). The UK Biobank replication cohort is a publicly available dataset for research purposes and can be accessed/applied from https://www.ukbiobank.ac.uk. The deCODE Genetics dataset summary results can be requested from the deCODE authors.
