# Supplementary material for: MEPE loss-of-function variant associates with decreased bone mineral density and increased fracture risk
Source: Nat Commun. 2020 Oct 23;11:4093. doi: 10.1038/s41467-020-17315-0 (PMC7585430; doi:10.1038/s41467-020-17315-0)
Supplement: Supplementary file 1 — Supplementary Information [file 41467_2020_17315_MOESM1_ESM.docx]

**Supplementary Information**

***MEPE* loss-of-function variant associates with decreased ultradistal forearm bone mineral density and increased fracture risk**

Surakka et al.

**This file contains:**

Supplementary Figures 1-3

Supplementary Tables 1-3

Supplementary Note

**Other Supplementary Materials for this manuscript include the following:**

Supplementary Data 1-3

**Supplementary Figures**

**Supplementary Figure 1. *MEPE* locus conditional zoom-plot in HUNT.**


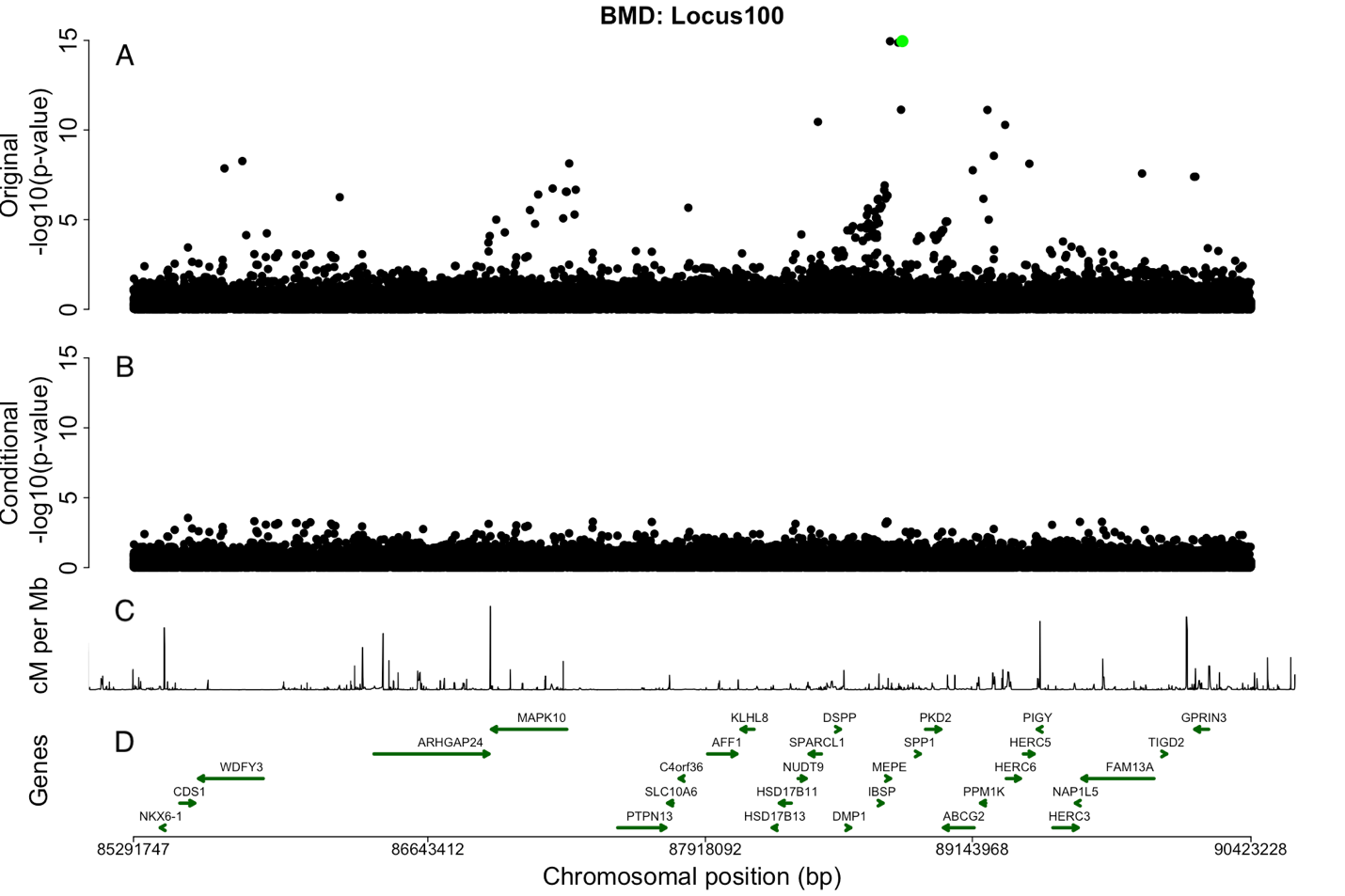


This figure of the MEPE locus in chromosome 4 demonstrates that the rare indel explains the association signal. The LoF indel variant, p.(Lys70IlefsTer26), has been highlighted with green in the upper panel (A) with the original association results. The second panel (B) represents the association results after adjusting for the LoF variant. The third panel (C) shows the recombination structure in the area and the last panel (D) shows the position genes.

**Supplementary Figure 2. Age trends in BMD for *MEPE* LoF mutation carriers**

In this figure we have compared the forearm bone mineral density (BMD) in the MEPE loss-of-function (LoF) mutation, p.Lys70IlefsTer26, carriers (darker boxes) compared to non-carriers (lighter boxes) in the HUNT dataset (N=19,705). The upper panel shows the results for males and lower for females both binned by age groups shown in X-axis. In each of the boxplots, the horizontal line in the middle of the box presents the median value and the lower and upper edges of the box 25^th^ and 75^th^ percentiles (1^st^ and 3^rd^ quartiles). The whiskers present either the smallest/largest value, or (in case of outliers are observed in the distribution, noted with rings) 1.5×the interquartile range.

**Supplementary Figure 3. Spearman correlation between effects in HUNT and published UKbb BMD results.**

The Y-axis of the figure is the Spearman correlation between the effect size in HUNT and in UKbb given the P-value threshold in UKbb = 1e-(x-axis value). For example, the Spearman correlation between the two studies is ~0.8 when restricting the calculation to SNPs with P-value < 1×10^-50^ in the UKbb dataset.

**Supplementary Tables**

**Supplementary Table 1. *MEPE* LoF variant p.(Lys70IlefsTer26) association to clinical end-points in the deCODE and UK Biobank datasets.** This table shows the association results for both replication datasets (UK Biobank and deCODE) for the clinical end-points related to bone mineral for the *MEPE* loss-of-function frameshift deletion, p.Lys70IlefsTer26 (rs753138805, chr4: 88766219 GAAA/-). The effect of a variant is presented with the odds ratio (OR) and the significance using the uncorrected two tailed Z-test (for log(OR)) P-value.

| End Point | Cohort | OR | N cases | N controls | Association test P-value |
| --- | --- | --- | --- | --- | --- |
| Any Fracture (over 40 year-old, low trauma, excluding hand, feet and skull) | deCODE | 1.73 | 20744 | 213027 | 0.082 |
| Any Fracture | deCODE | 1.27 | 95622 | 280362 | 0.279 |
| Forearm Fracture (over 40 year-old, low trauma) | deCODE | 3.39 | 4671 | 162091 | 0.005 |
| Forearm Fracture | deCODE | 1.89 | 19086 | 322857 | 0.048 |
| Hip Fracture (over 50 year-old) | deCODE | 1.34 | 9523 | 169038 | 0.517 |
| Vertebral Fracture (over 40 year-old, low trauma) | deCODE | 1.70 | 1301 | 165461 | 0.515 |
| Osteoporosis (over 40 year-old, primary diagnosis) | deCODE | 1.22 | 2633 | 172703 | 0.755 |
| Any Fracture (ICD codes S22 - S92, excluding skull [S02] and neck [S12]) | UK Biobank | 1.76 | 14073 | 260943 | 0.050 |

OR: Odds ratio

N: number of

**Supplementary Table 2. Fracture rate and mean forearm BMD with clinical and genetic predictors in the subset with measured BMD in HUNT.** In this table, the significance of different predictors for fractures is shown using the odds ratio (OR) and 95% confidence interval (CI) together with the two tailed uncorrected Fisher-test P-values.

| Predictor | BMD mean (SD) mg/dl | Fracture OR [95% CI] | Fisher test P-value | Fracture prevalence |
| --- | --- | --- | --- | --- |
| Low BMD (<-2.5 SD units) | 0.121 (0.015) | 1.65 [1.15; 2.35] | 0.007189 | 53.3% |
| Low BMD polygenic score (<1%) | 0.400 (0.099) | 0.89 [0.66; 1.19] | 0.4602 | 38.2% |
| Low BMD polygenic score (<5%) | 0.400 (0.101) | 0.98 [0.86; 1.12] | 0.7898 | 40.6% |
| Low BMD polygenic score (<10%) | 0.398 (0.104) | 1.04 [0.94; 1.14] | 0.4378 | 41.8% |
| *MEPE* LoF | 0.373 (0.098) | 1.39 [1.11; 1.74] | 0.004747 | 49.0% |
| whole sample | 0.396 (0.102) | NA |  | 41.0% |

BMD: Bone mineral density

SD: standard deviation

OR: Odds ratio

CI: Confidence interval

LoF: Loss-of-function

**Supplementary Table 3. Clinical characteristics of HUNT individuals who carry the rare and common genetic variants at *MEPE* relative to the population.** This table present characteristics of the HUNT study set as a whole, together with different subsets defined by carried genetic mutations.

|  |  |  |  |  |
| --- | --- | --- | --- | --- |
| **Measurement/Phenotype (statistics)** | **Whole dataset** | ***MEPE* common rs1471403 risk allele heterozygotes** | ***MEPE* common rs1471403 risk allele homozygotes** | ***MEPE* LoF allele carriers** |
| Birthyear Mean (SD) in years Number of samples | 1950.1 (18.4) 69635 | 1950.0 (18.4) 29739 | 1950.3 (18.4) 33024 | 1948.8 (19.0) 946 |
| Sex %Males / %Females Number of samples | 53.0 / 47.0. 69635 | 53.0 / 47.0. 29739 | 53.0 / 47.0 33024 | 52.5 / 47.5 946 |
| Bone mineral density Mean (SD) in g/cm2 Number of samples | 0.40 (0.10) 19705 | 0.40 (0.10) 8486 | 0.40 (0.10) 9301 | 0.37 (0.10) 304 |
| Percentage with fractures 95% Confidence interval Number of samples | 34.7% [34.3; 35.1] 69635 | 34.8% [34.3; 35.3] 29739 | 34.9% [34.4; 35.4] 33204 | 42.5% [39.3; 45.7] 946 |
| Percentage with osteoporosis 95% Confidence interval Number of samples | 11.6% [11.4; 11.8] 68552 | 11.7% [11.3; 12.1] 29267 | 11.7% [11.4; 12.0] 32683 | 16.2% [13.8; 18.9] 932 |
| LoF: Loss-of-function  SD: standard deviation | |  |  |  |

**Supplementary Note**

**Regeneron Genetics Center Banner Author List and Contribution Statements**

All authors/contributors are listed in alphabetical order.

***RGC Management and Leadership Team***

Goncalo Abecasis, Ph.D., Aris Baras, M.D., Michael Cantor, M.D., Giovanni Coppola, M.D., Aris Economides, Ph.D., Luca A. Lotta, M.D., Ph.D., John D. Overton, Ph.D., Jeffrey G. Reid, Ph.D., Alan Shuldiner, M.D.

Contribution: All authors contributed to securing funding, study design and oversight. All authors reviewed the final version of the manuscript.

***Sequencing and Lab Operations***

Christina Beechert, Caitlin Forsythe, M.S., Erin D. Fuller, Zhenhua Gu, M.S., Michael Lattari, Alexander Lopez, M.S., John D. Overton, Ph.D., Thomas D. Schleicher, M.S., Maria Sotiropoulos Padilla, M.S., Karina Toledo, Louis Widom, Sarah E. Wolf, M.S., Manasi Pradhan, M.S., Kia Manoochehri, Ricardo H. Ulloa.

Contribution: C.B., C.F., K.T., A.L., and J.D.O. performed and are responsible for sample genotyping. C.B, C.F., E.D.F., M.L., M.S.P., K.T., L.W., S.E.W., A.L., and J.D.O. performed and are responsible for exome sequencing. T.D.S., Z.G., A.L., and J.D.O. conceived and are responsible for laboratory automation. M.P., K.M., R.U., and J.D.O are responsible for sample tracking and the library information management system.

***Genome Informatics***

Xiaodong Bai, Ph.D., Suganthi Balasubramanian, Ph.D., Leland Barnard, Ph.D., Andrew Blumenfeld, Gisu Eom, Lukas Habegger, Ph.D., Young Hahn, Alicia Hawes, B.S., Shareef Khalid, Jeffrey G. Reid, Ph.D., Evan K. Maxwell, Ph.D., William Salerno, Ph.D., Jeffrey C. Staples, Ph.D., Ashish Yadav, M.S.

Contribution: X.B., A.H., W.S. and J.G.R. performed and are responsible for analysis needed to produce exome and genotype data. G.E., Y.H., and J.G.R. provided compute infrastructure development and operational support. S.K., S.B., and J.G.R. provide variant and gene annotations and their functional interpretation of variants. E.M., L.B., J.S., A.B., A.Y., L.H., J.G.R. conceived and are responsible for creating, developing, and deploying analysis platforms and computational methods for analyzing genomic data.

***Clinical Informatics***

Michael Cantor, M.D., Deepika Sharma, DHMS

Contribution: DS and MC contributed development of clinical phenotypes for analysis.

***Analytical Genomics and Data Science***

Goncalo Abecasis, Ph.D., Joshua Backman, Manuel Allen Revez Ferreira, Jack Kosmicki

Contribution: GA, JB, MF and JK contributed to all analytical genetics and association analyses.

***Research Program Management***

Marcus B. Jones, Ph.D., Lyndon J. Mitnaul, Ph.D.

Contribution: All authors contributed to the management and coordination of all research activities, planning and execution. All authors contributed to the review process for the final version of the manuscript.
