# Supplementary material for: MEPE loss-of-function variant associates with decreased bone mineral density and increased fracture risk
Source: Nat Commun. 2020 Oct 23;11:4093. doi: 10.1038/s41467-020-17315-0 (PMC7585430; doi:10.1038/s41467-020-17315-0)
Supplement: Supplementary file 3 — Description of Additional Supplementary Files [file 41467_2020_17315_MOESM3_ESM.docx]

**Description of Additional Supplementary Files**

**File name:** Supplementary Data 1

**Description:** HUNT association results for previously published variants at the MEPE locus before and after conditioning on p.(Lys70IlefsTer26)

**File name:** Supplementary Data 2

**Description:** PheWAS in HUNT for bone-related traits and MEPE LoF variant p.(Lys70IlefsTer26)

**File name:** Supplementary Data 3

**Description:** Original and HUNT association results for previously published independent variants associated with BMD
